# Drug-Coated Balloons Beyond In-Stent Restenosis

**DOI:** 10.31083/RCM45108

**Published:** 2026-02-25

**Authors:** Rahul Gupta, Chandrashekar Bohra, Krishna Santosh Vemuri, Gaurav Arora, Amit Gulati, Isha Ranadive, Samin K. Sharma, Amit Hooda

**Affiliations:** ^1^Department of General Internal Medicine, Cedars-Sinai Medical Center, Los Angeles, CA 90048, USA; ^2^Cardiovascular Institute of Central Florida/HCA Ocala Hospital, Ocala, FL 34471, USA; ^3^Division of Cardiology, Mount Sinai Fuster Heart Hospital, New York, NY 10029, USA; ^4^Division of Cardiology, Mount Sinai Beth Israel, New York, NY 10029, USA

**Keywords:** drug-coated balloon, drug-eluting balloon, drug-eluting stent, dual antiplatelet therapy

## Abstract

Drug-coated balloons (DCBs) are rapidly gaining prominence, owing to the associated expanding clinical applications and therapeutic potential in interventional cardiology. Moreover, a growing body of evidence from randomized trials, observational studies, and meta-analyses supports the efficacy of DCBs across a broad spectrum of coronary lesions, positioning these devices as a viable, stent-free alternative to conventional strategies. DCBs aim to lower the risks inherent to stent implantation, such as stent thrombosis and in-stent restenosis (ISR), and to enable abbreviated dual antiplatelet therapy in high-bleeding-risk (HBR) patients. Although drug-eluting stents (DESs) remain the current standard in percutaneous coronary intervention, DCBs are emerging as a novel and promising option in diverse clinical settings beyond ISR, including *de novo* lesions in both small and large vessels, bifurcation disease, patients with a high risk of bleeding, and even acute coronary syndromes. This article aims to provide a comprehensive review of the evolving role, evidence base, and expanding indications of DCB-related therapy beyond ISR.

## 1. Introduction 

The growing burden of coronary artery disease has resulted in millions of individuals undergoing percutaneous coronary intervention (PCI) each year, often involving the implantation of one or more drug-eluting stents (DESs) [1, 2]. Current data suggest that in-stent restenosis (ISR) occurs within the first year in approximately 5%–10% of patients who undergo PCI with DES in the United States [3]. Traditionally, ISR has been managed with balloon angioplasty followed by implantation of an additional DES, when feasible. However, this approach can increase the risk of long-term restenosis and change the patient’s surgical coronary artery bypass graft (CABG) candidacy based on the location of DES implantation [4].

Over the past decade, DCBs have emerged as a viable alternative to routine angioplasty and to DES in diverse clinical scenarios. In 2018, the European Society of Cardiology included drug-coated balloons (DCBs) in its revascularization guidelines with a Class I recommendation for the treatment of coronary ISR [5]. These devices have also seen increasing adoption across the Asia-Pacific region and Latin America for both ISR and previously untreated small-vessel coronary disease. The multicenter, randomized AGENT IDE trial evaluated the efficacy of a paclitaxel-coated balloon versus an uncoated balloon in treating ISR, enrolling 600 patients. At a 12-month follow-up, the DCB group demonstrated a significantly lower rate of target lesion failure (TLF) compared to the routine balloon angioplasty group (17.9% vs. 28.6%; *p* = 0.003) [6]. These findings led to the United States (US) Food and Drug Administration (FDA) approval of paclitaxel-coated DCBs for the treatment of coronary ISR on March 1, 2024.

Neointimal hyperplasia and/or neo-atherosclerosis are proposed mechanisms that can lead to ISR despite advances in the DES technology [7]. This can cause recurrence of acute-on-chronic or acute coronary syndromes, thereby requiring or mandating the need for revascularization. The proposed mechanism of DCB is based on the concept of delivering an antiproliferative agent to prevent restenosis [8]. Though balloon catheters coated with different antiproliferative agents were tested, the taxane group of compounds, in particular paclitaxel, showed promising results [9]. This concept was first tested in a trial named Treatment of In-Stent Restenosis by Paclitaxel-Coated Balloon Catheters (PACCOCATH ISR), the results of which were published in November 2006. The results were promising and showed a reduction in ISR by local drug delivery [10]. The additional positive effect was luminal increase, observed later, described as a positive remodeling phenomenon [11]. The use of a highly lipophilic compound as a local antiproliferative agent combined with a contrast medium as an excipient ensured rapid drug delivery in a short contact period [10]. A meta-analysis by Zhang *et al*. [12] done for DCB use in *de novo* coronary artery lesions reaffirmed that the DCB-only strategy is superior to traditional angioplasty and comparable with DES. The introduction of DCB has ushered in a new era in the management of coronary artery disease (CAD), offering patients an effective stent-free alternative that eliminates the associated maladaptive biological responses.

## 2. Methods

A comprehensive literature search was conducted in PubMed from February 2002 to October 2025 to identify relevant studies evaluating the safety, efficacy, and clinical outcomes of DCBs in coronary interventions. The search strategy incorporated combinations of keywords and Medical Subject Headings (MeSH) terms such as “drug-coated balloon”, “drug-eluting balloon”, “coronary artery”, and “clinical outcomes.” Eligible publications included randomized controlled trials, observational studies, registries, and meta-analyses published in English, with full-text availability. Abstract-only publications and case reports were excluded.

Although this narrative review attempted to capture all relevant DCB studies, the methodology has inherent limitations, including reliance on a single database (PubMed) and the absence of a formal risk-of-bias assessment.

## 3. *De Novo* Lesions

### 3.1 Large Coronary Vessels (≥2.75 mm)

The use of DCB for *de novo* lesions in large coronary vessels is gaining widespread traction, supported by growing clinical experience and emerging evidence. The third report of the International DCB Consensus Group echoed this evolving paradigm, endorsing the expanding role of DCBs in this setting [13]. A DCB-only PCI strategy appears to be safe and effective across diverse lesion subsets, including *de novo* large coronary lesions [14, 15, 16, 17], ostial lesions [18], bifurcations [15], ST-elevation myocardial infarction (STEMI) [19], and may facilitate reductions in both the duration of dual antiplatelet therapy (DAPT) and the overall extent of drug-eluting stent implantation. Multiple clinical trials have demonstrated consistently favorable outcomes with DCB use in *de novo* large-vessel disease (Table 1, Ref. [14, 15, 16, 17, 18, 19, 20, 21]).

**Table 1.** **Summary of clinical trials/studies of DCB in *de novo* large coronary lesions**.

Study/Trial Name	Total no. of patients (n)	Study design	Treatment arm	Clinical follow-up	Conclusion
Uskela *et al*. 2019 [17]	487 (60% >3mm)	Single-center all-comers retrospective registry	DCB (Sequent Please) in *de novo* lesions with the possibility of bailout stent in stable CAD/ACS patients	TLR 1.4% in stable CAD, 2.8% in ACS @ 12 months	PCI using a DCB-only strategy is a safe and efficient approach in large *de novo* vessels
Yu *et al*. 2019 [16]	200	Single-center retrospective registry	DCB (Sequent Please) in *de novo* lesions for large vessels, small vessel DCB only PCI	TLR of 0% @ 10 months	PCB is safe and effective for coronary lesions in diameters greater than 2.8 mm
Hu *et al*. 2022 [15]	119	Multicenter prospective observational study	DCB (Sequent Please or Swide)	TLR of 3.4% @ 2 years	DCB is safe for bifurcation and non-bifurcation lesions
Gitto *et al*. 2023 [20]	848	Retrospective observational propensity-matched study	DCB (Magic Touch or Selution or IN.PACT or RESTORE) vs second-generation DES	TLR 4.1% (DCB) vs 9.8% (DES); *p* = 0.15 @ 2 years	DCB in LAD reduces the stent length burden and TLF
Pan *et al*. 2023 [18]	397	Retrospective observational propensity-matched study	DCB (Sequent Please) vs second-generation DES	TLR 4.9% (DCB) vs 16.33% (DES); *p* = 0.008 @ 2 years	DCB is a safe alternate strategy in ostial LAD and LCx lesions
Merinopoulos I *et al*. 2023 [19]	1139	Single-center retrospective propensity-matched study	DCB (Paclitaxel-coated) vs second-generation DES	TLR 0.2% (DCB) vs 0.7% (DES) at 30 days and no difference @ year (*p* = 0.41)	DCB-only angioplasty is safe in STEMI patients
Gao C *et al*. 2024 [21]	2272	Open-label randomised non-inferiority control trial	DCB (Swide) vs DES (SES) with *de novo* non-complex lesions	DoCE: 6.4% (DCB) vs 3.4% (DES); *p* = 0.0008 @ 24months	DCB group failed to achieve non-inferiority
Gobbi *et al*. 2025 [14]	2114	Systematic review and meta-analysis	DCB vs DES	TLR of 4.3% (DCB) vs 6.9% (DES); *p* = 0.059 @ 2 years	DCB use in large coronary vessels is safe and effective

DES-SES, drug-eluting stent-sirolimus-eluting stent; PCB, paclitaxel-coated balloons; DCB, drug-coated balloon; TLR, target lesion revascularization; CAD, coronary artery disease; PCI, percutaneous coronary intervention; LAD, left anterior descending; TLF, target lesion failure; LCx, left circumflex; STEMI, ST-elevation myocardial infarction; DoCE, device-oriented composite endpoint; ACS, acute coronary syndrome.

The study by Uskela *et al*. [17] investigated the efficacy and safety of DCB-only PCI in patients presenting with stable angina and acute coronary syndromes. In a cohort of 487 patients, more than half of them presented with acute coronary syndrome, and more than 60% of lesions were in >3 mm vessels. The rate of ischemia-driven revascularization was low in both stable CAD (1.4% at 12 months) and acute coronary syndrome (2.8% at 12 months) patients, with no difference between the DCB and DES groups (log rank *p* = 0.136). Similarly, the studies by Hu *et al*. [15] and Yu *et al*. [16] demonstrated the safety and feasibility of DCB in large *de novo* coronary lesions.

Gitto *et al*. [20] studied a DCB-based approach, either a stand-alone or hybrid approach, in left anterior descending (LAD) PCI. They demonstrated that DCB-based treatment is associated with reduced stent burden and a lower risk of target lesion failure at 2 years compared with DES-only PCI (DCB, 4.1% vs DES, 9.8%; hazard ratio (HR) 0.51, 95% CI 0.20–1.27; *p* = 0.15), mainly driven by less target lesion revascularization. Pan *et al*. [18] demonstrated the safety and feasibility of a DCB-based approach in ostial LAD and ostial left circumflex (LCx) lesions at 2 years of follow-up. Target lesion revascularization (TLR) occurred in 4.9% in the DCB group and 16.33% in the DES group at 2 years (odds ratio (OR) 0.264, CI 0.093–0.752; *p* = 0.008).

Furthermore, Gobbi *et al*. [14] conducted a comprehensive systematic review and meta-analysis for *de novo* lesions in large coronary vessels (>2.75 mm), including studies that utilized DCB-only or hybrid angioplasty. The study included 15 studies [14 non-randomized controlled trials and 1 randomized controlled trial (RCT)] that showed that DCB was associated with a lower rate of TLR when compared to patients treated with DES, even though statistical significance was not reached, there was a trend towards significance (4.3% vs 6.9%, OR = 0.71, CI 0.49–1.01, *p* = 0.059). At a mean follow-up of 20.6 + 1.9 months, the TLR, which is the primary outcome, occurred at 4%. The key secondary endpoints were cardiac death, myocardial infarction, and target lesion failure that occurred at rates of 3.5%, 5.7%, and 5.3%, respectively. There were no differences in death or myocardial infarction (MI), with less frequent TLF occurrence in DCB-treated patients [14].

The landmark open-label randomized controlled trial from China, REC-CAGEFREE I, failed to demonstrate non-inferiority of DCB (Swide DCB; Shenqi) angioplasty with rescue stenting compared with intended sirolimus-eluting DES (Firebird2; MicroPort) for patients with *de novo*, non-complex coronary artery lesions. The study included 2272 patients. Over 2 years of follow-up, a device-oriented composite endpoint (DoCE)-incorporating cardiac death, target-vessel MI, and clinically and physiologically indicated TLR- occurred in 6.4% with the DCB group and 3.4% with the DES group (upper boundary of the one-sided 95% CI 4.52; *p*_non-inferiority_ = 0.65; two-sided 95% CI 1.27–4.81; *p* = 0.0008) [21].

However, the most recent 3-year findings from REC-CAGEFREE I showed that the overall difference in DoCE was mainly driven by the clinically and physiologically indicated target lesion revascularization (CPI-TLR) (4.2% vs 1.6%; HR 2.66; 95% CI 1.54–4.57), mostly elective revascularizations. No differences were observed in cardiovascular death or target-vessel MI. The landmark analyses showed the absolute increase in DoCE progressively narrowed over time: 1.69% from 0 to 1 year, 1.1% from 1 to 2 years, and 0.58% from 2 to 3 years (*p* for trend = 0.023). Additionally, a trend towards less bleeding with DCB vs DES was noted (1.4% vs 2.6%; HR 0.55; 95% CI 0.3–1.02) [22].

It is very important to recognize several key limitations associated with this study to enable better interpretation of its findings. First, the lesions included were non-complex—typically short and in non-small-diameter vessels—an anatomic subset that already demonstrates excellent outcomes with contemporary DES. As such, these lesions do not reflect the clinical scenarios in which DCBs might offer greater benefit. DCB therapy may be particularly advantageous in settings where DES performance remains suboptimal, such as in small vessels, diffuse disease, complex anatomies, and diabetes mellitus.

Second, because sirolimus-coated balloons were not commercially available in China, all patients were treated with paclitaxel-coated balloons. Therefore, the results should not be extrapolated to the sirolimus-coated balloons. Moreover, the specific DCB used in the study (Swide DCB) is not globally approved and employs a paclitaxel-iopramide formulation (paclitaxel of 3 µg/mm^2^). Caution is warranted in assuming class effect across other paclitaxel-coated balloons.

Additional limitations include the underrepresentation of females (30% of the cohort) and the fact that the study population consisted exclusively of East Asian patients, limiting generalizability to other racial and ethnic groups. Furthermore, the use of intravascular imaging was low, given non-complex lesions (10.3%) [22].

### 3.2 Small Coronary Vessels (≤2.75 or <3 mm)

Small-vessel coronary disease (SVD) continues to pose a therapeutic challenge despite advances in percutaneous coronary intervention. While DES has demonstrated efficacy in treating *de novo* lesions in small-caliber vessels, it is associated with a higher risk of late lumen loss and restenosis, often necessitating repeat revascularization [23]. DCB therapy presents a promising alternative to DES for managing this challenging subset of patients. Multiple studies have demonstrated outcomes with DCB that are similar to or even superior to those with DES in SVD (Table 2, Ref. [24, 25, 26, 27, 28, 29, 30, 31, 32]).

**Table 2.** **Summary of trials/studies of DCB in *de novo* small-vessel CAD**.

Study/Trial Name	Total no. of patients (n); Reference vessel size	Study design	Treatment arm	Clinical follow-up	Conclusion
PICCOLETO. 2010 [26]	60; ≤2.75 mm	RCT	Dior PCB vs Taxus DES	MACE 35.7% (PCB) vs 13.8% (DES); *p* = 0.054 @ 9 months	PCB failed to show equivalence to Taxus DES
BELLO. 2012 [27]	182; <2.8 mm	RCT	DCB (IN. PACT FALCON) vs Paclitaxel-eluting stents	TLR 4.4% (DCB) vs 7.6% (DES); *p* = 0.37 @ 6 months	Paclitaxel DCB associated with less angiographic late loss and similar rates of restenosis and revascularization
Funatsu *et al*. 2017 [30]	135; ≥2.0 and <2.75 mm	RCT	PCB (Sequent Please) vs POBA	TVF between PCB vs POBA (3.4 vs. 10.3%; *p* = 0.20) @ 24 weeks	PCB was not able to demonstrate superiority to POBA
RESTORE SVD China. 2018 [29]	230; ≥2.25 and ≤2.75 mm	RCT	DCB (RESTORE Paclitaxel) vs DES (RESOLUTE Zotarolimus)	TLF 4.4% (DCB) vs 2.6% (DES) @ 12 months	Restore DCB was noninferior to the RESOLUTE DES
BASKET-SMALL 2. 2020 [24, 28]	758; <3 mm	RCT	DCB (Sequent Please) vs second-generation DES	MACE 15% (DCB) vs 15% (DES); p = 0.95 @ 3 years	DCB was non-inferior to DES
BIO-RISE CHINA. 2022 [31]	212; 2.0–2.75 mm	Prospective trial	BCB (Biolimus) vs POBA	TLF 6.7% (BCB) vs 13.9% (POBA) @ 12 months	BCB showed superior efficacy over plain balloon angioplasty
PEPCAD China SVD. 2023 [32]	270; 2.0–2.75 mm	Prospective multicenter trial	DCB (SeQuent Please) vs POBA	LLL 0.10 ± 0.33 mm DCB vs 0.25 ± 0.38 mm with POBA (*p* = 0.0027).	DCB showed lower late luminal loss than POBA
				TLR 3.9% (DCB) vs 6.9% (POBA); *p* = 0.362 @ 9 months	
PICCOLETO II. 2023 [25]	232; ≤2.75 mm	RCT	DCB (Elutax SV) vs DES (DES-E)	MACE 10.8% (DCB) vs 20.8% (DES); *p* = 0.046 @ 3 years	Lower risk of MACE with DCB at 3 years

TVF, target vessel failure; DES-E, drug-eluting stent everolimus; BCB, biolimus-coated balloon; HBR, high bleeding risk; RCT, randomized controlled trial; MACE, major adverse cardiac events; POBA, plain old balloon angioplasty; LLL, late luminal loss; SV, small vessel.

The long-term follow-up is available for three trials. The three-year follow-up of the BELLO trial [33], which included 173 patients, showed a statistically significant difference in major adverse cardiac events (MACE) between paclitaxel-coated balloons and DES (paclitaxel) (14.4% vs 30.4%, *p* = 0.015). There were no differences between the groups in TLR and TLF rates. The BASKET-SMALL 2, multicenter trial randomized 758 patients (DCB = 382 vs. DES = 376), comparing DES (28% paclitaxel eluting stents and the rest of everolimus eluting) with a paclitaxel-iopromide-coated DCB, demonstrating the noninferiority of DCB-treated patients to the DES groups in *de novo* small vessel disease at 3 years, with similar MACE between the groups 15% (DCB) vs. 15% (DES) HR: 0.99; *p* = 0.95. Rates of probable or definite vessel or stent thrombosis (Kaplan–Meier estimate 1% vs. 2%; HR 0.33, 95% CI 0.07–1.64; *p* = 0.18) and major bleeding (Kaplan–Meier estimate 2% vs. 4%; HR 0.43, 95% CI 0.17–1.13; *p* = 0.088) were numerically lower in the DCB group compared with the DES group, although these differences did not reach statistical significance [24].

The PICCOLETO II trial demonstrated that DCB was superior to everolimus-eluting stents in small vessel coronary artery disease at both 1-year and 3-year follow-up. The primary outcome of in-lesion late lumen loss at 6 months was 0.04 mm in the drug-coated balloon group compared with 0.17 mm in the drug-eluting stent group (*p* for noninferiority = 0.01, *p* for superiority = 0.03). At 3 years, MACE rates were significantly lower in the DCB group (10.8% vs 20.8%, *p* = 0.046), and TLR rates, although lower in the DCB group, did not reach statistical significance (8.8% vs 14.8%, *p* = 0.18) [25].

Even though the initial randomized controlled trial (RCT) PICCOLETO [26] failed to show efficacy over DES, subsequent trials, including BELLO [27], BASKET SMALL 2 [28], PICCOLETO II [25], and RESTORE SVD [29], established non-inferiority and lower late luminal angiographic loss than DES at 6 months to 3 years of follow-up. The multicenter, prospective, randomized controlled trial by Funatsu *et al*. [30] showed significantly lower late luminal loss (LLL) in the PCB group; however, this study was unable to demonstrate superiority over POBA. Further trials like BIO-RISE CHINA [31], PEPCAD CHINA SVD [32] established the superior efficacy of DCB over plain old balloon angioplasty (POBA) (Table 2).

A large meta-analysis by Felbel *et al*. [23], comprising 37 studies and 31,385 patients (28,147 with DES vs. 3299 with DCB), demonstrated similar clinical and angiographic outcomes in patients with coronary small vessel lesions with a reference diameter of ≤3.0 mm. The MACE rate was 4% and 9% in the DCB and DES groups, respectively [0.09 (0.07–0.10) vs. 0.04 (0.02–0.08)], while TLR was 4% in both groups [0.04; 95% CI 0.03–0.05 (DES) vs. 0.03–0.07 (DCB)] [23].

Another recent meta-analysis of 29 RCTs involving more than 8000 patients with small vessel disease demonstrated no significant differences in clinical outcomes between DCB and newer generation DES [34].

## 4. Bifurcation/Side Branch Lesions 

**Fig. 1.**
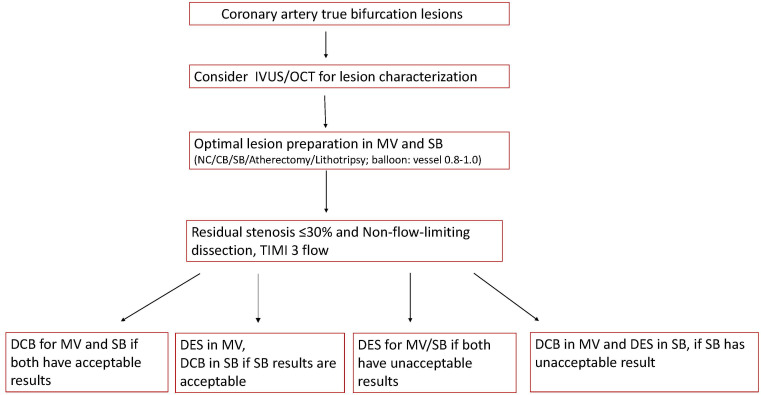
**An expert opinion-based practical algorithm for the use of DCB in coronary bifurcation lesions [37, 38]**. MV, main vessel; TIMI, thrombolysis in myocardial infarction; NC, noncompliant balloon; SB, side branch; IVUS, intravascular ultrasound; OCT, optical coherence tomography.

Percutaneous coronary intervention for bifurcation lesions remains technically demanding and is associated with increased procedural complexity and adverse outcomes [35]. The two-stent strategy, while sometimes necessary, carries a higher risk of complications compared to the single-stent approach. The European Society of Cardiology (ESC) [5] and the European Bifurcation Club (EBC) [36] both advocate for a main branch-only stenting approach with provisional side-branch stenting for bifurcation lesions. However, the optimal strategy for managing bifurcation lesions using DCB continues to evolve (Fig. 1, Ref. [37, 38]).

DCB therapy offers a promising alternative by potentially simplifying the procedure, avoiding stent jailing, preserving side branch patency, and reducing procedural time, particularly in cases where a two-stent technique might otherwise be considered. Based on multiple trials, the use of DCBs in bifurcate lesions is appealing (Table 3, Ref. [39, 40, 41, 42]).

**Table 3.** **Summary of clinical trials/studies of DCB use in bifurcation disease**.

Study/Trial name	Total no. of patients (n)	Study design	Treatment arm	Clinical follow-up	Conclusion
Megaly *et al*. 2018 [39]	349	Meta-analysis	DCB vs POBA	No difference in TLR (OR, 0.85; 95% CI, 0.3–2.4; *p* = 0.76)	DCB is associated with lower SB late lumen loss with no difference in clinical outcomes
BEYOND. 2020 [42]	222	RCT	PEB vs BA	PEB (28.7% ± 18.7%) vs BA (40.0% ± 19.0%) target lesion stenosis *p*_Superiority_ <0.0001) @ 9 months	PEB had better angiographic results at 9 months, with no difference in clinical events
Corballis *et al*. 2021 [40]	-	Systematic review and focused meta-analysis	DCB vs POBA/DES		DCB is associated with improved late lumen loss when compared with DES or POBA
DCB-BIF. 2025 [41]	784	RCT	DCB (Paclitaxel-coated balloon) vs NCB, for the side branch	MACE 7.2% (DCB) vs 12.5% (NCB); *p* = 0.013 @ 1year	DCB for the compromised side branch was superior to NCB angioplasty

NCB, non-compliant balloon; PEB, paclitaxel-eluting; BA, balloon angioplasty.

A comprehensive meta-analysis by Megaly *et al*. [39] included four studies (three RCTs and one observational study; BABILON [43], PEPCAD BIF [44], DEBUIT [45], and Herrador *et al*. [46]) that enrolled 349 patients with a mean follow-up of 15.1 ± 5.8 months. There was no difference in the risk of TLR between DCB and uncoated balloon angioplasty (BA) (OR, 0.85; 95% CI, 0.3–2.4; *p* = 0.76). In a sensitivity analysis including only RCTs, DCB and BA still had a similar risk of TLR (OR, 1.1; 95% CI, 0.24–4.93; *p* = 0.91).

Corballis *et al*. [40] revealed that DCBs can be used safely in main branch (MB) bifurcation lesions with a low rate of late lumen loss in the side branch as compared to drug-eluting stents and traditional balloon angioplasty (mean difference = 0.24 mm; *p* = 0.01) [41]. Similar findings were suggested by another meta-analysis involving 5 RCTs and 5 non-randomized observational studies, wherein DCB was found to be effective in protecting *de novo* coronary bifurcation lesions at short- and medium-term follow-up. The MACE rate of the DCB group was strikingly lower than that of the non-DCB group after a 9-month follow-up period [OR = 0.21, 95% CI (0.05, 0.84), *p* = 0.03] [35].

The recently published study, DCB BIF, a large, adequately powered randomized controlled trial, compared DCB with a non-compliant balloon in true simple coronary bifurcation lesions. The study enrolled 784 patients, which revealed the primary end point (composite of cardiac death, target vessel myocardial infarction, or clinically driven target-lesion revascularization at the 1-year follow-up) occurred less frequently in the DCB group when compared to the non-compliant balloon (7.2% vs 12.5%; HR: 0.56; 95% CI: 0.35–0.88; *p* = 0.013), driven by a reduction in myocardial infarction [41].

Emerging data suggest that DCBs are gradually being adopted as a viable alternative for treating side branch bifurcation lesions, demonstrating reduced late lumen loss in side branches compared to plain old balloon angioplasty (POBA) or DES (Table 3).

The second report of the Asia-Pacific Consensus Group proposed a structured strategy for the use of DCBs in true bifurcation lesions [37]. Combining DCB and DES yielded good results for the left main bifurcation lesions, while DCB-alone treatment for the main vessel only was supported for bifurcation lesions in the Korean Optical Coherence Tomography study [37].

## 5. High Bleeding Risk 

High bleeding risk patients undergoing PCI remain a particularly challenging subset, especially elderly individuals and those on oral anticoagulants (OAC). Bleeding in contemporary PCI increases the risk of mortality and morbidity, prolongs hospital stay, and adds to overall healthcare costs. Although the duration of DAPT has been shortened to 1–3 months with newer-generation DES, DCB use may further offer the advantage of early DAPT discontinuation- often within <1 month in selected cases with careful risk tradeoffs [47]. Contemporary studies have shown that DCB-only PCI can be performed safely with single antiplatelet therapy in this high-risk population, without a significant increase in thrombotic risk [48, 49]. Studies of DCBs indicate a low risk of vessel thrombosis due to the absence of a permanent metal scaffold, thereby benefiting patients at high risk of bleeding and further enabling the early discontinuation of antiplatelet therapy [50].

Although the DEBUT trial demonstrated the superiority of DCB over BMS in *de novo* coronary lesions among high-bleeding risk patients, these results are no longer applicable in contemporary practice, as BMS are no longer used [51].

A prespecified subgroup analysis of the BASKET-SMALL 2 trial in the high-bleeding-risk (HBR) population demonstrated a trend toward reduced severe bleeding with DCB (shorter DAPT) compared with DES PCI on standard DAPT [28].

A retrospective study, in which approximately half of the patients had at least one bleeding risk factor, demonstrated the safety of DCB use in both ACS and stable CAD populations, with a mean DAPT duration of 1–3 months [17]. The RESTORE SVD China study also demonstrated non-inferiority of a DCB strategy compared with DES for *de novo* SVD, with respect to angiographic restenosis, MACE, and repeat revascularization [29]. Furthermore, similar findings were observed in STEMI patients in the REVELATION trial, which demonstrated the non-inferiority of DCB compared with DES, while maintaining the standard antiplatelet therapy duration recommended for DES [52].

Adequately powered RCTs randomizing HBR patients to DCB-only (with predefined bailout stenting) vs contemporary second-generation DES, with bleeding and ischemic endpoints, and prespecified DAPT strategies are required. Ongoing trials such as DCB-HBR (Drug-Coated Balloon Versus Drug-Eluting Stent for Treatment of *De novo* Coronary Lesions in Patients with High Bleeding Risk) and DEBATE (Drug-Coated Balloon in Anticoagulated and Bleeding Risk Patients Undergoing PCI) aim to address this knowledge gap [37].

Despite promising evidence from prior studies, the optimal composition and duration of antiplatelet therapy following DCB-only PCI has yet to be established and continues to evolve as new data emerge. Table 4 (Ref. [37, 48, 49, 51, 52]) summarizes current recommendations for antiplatelet therapy after DES and DCB-only interventions across various clinical scenarios, based on the best available evidence. Few observational studies have demonstrated 1-month single antiplatelet therapy (SAPT) with DCB-only PCI to be safe and efficacious primarily in stable CAD; however, a small number of ACS patients were also included in these studies [48, 49].

**Table 4.** **Summary of current recommendations for dual and single antiplatelet therapy after DES and DCB use across different clinical scenarios**.

Clinical Scenario	DES PCI	DCB-only PCI
ACS [52]	∼12 months	∼12 months
*De novo* lesions [37]	6 months	1–3 months
High risk bleeding [51]	1–3 months, Transition to SAPT	1 month, SAPT* [48, 49]

*Selected cases with careful risk assessment. SAPT, single antiplatelet therapy.

## 6. Acute Coronary Syndrome 

**Fig. 2.**
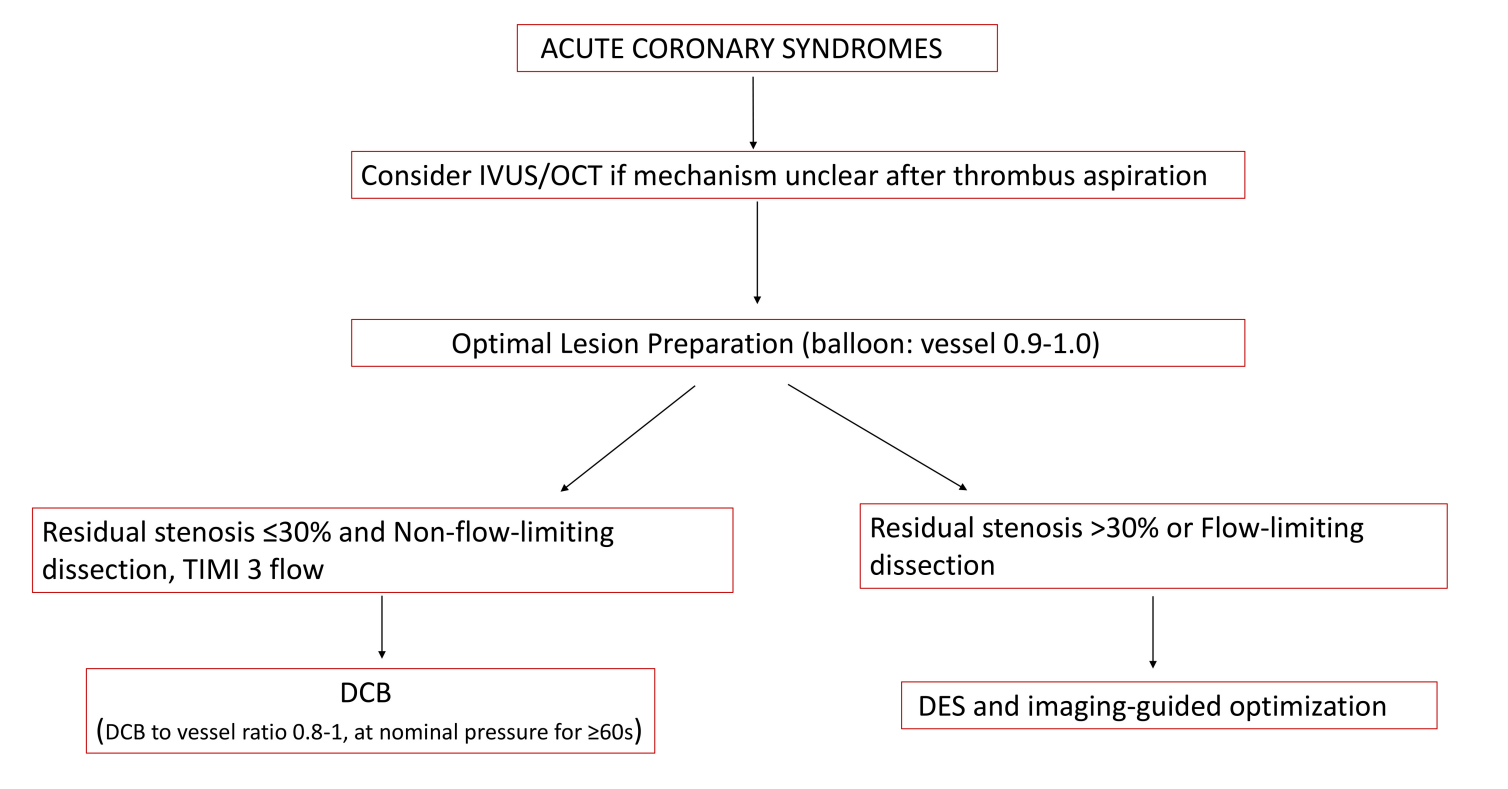
**An expert opinion-based practical algorithm for the use of DCB in ACS [37, 38]**.

Despite significant advancements in pharmacotherapy and stent technology, the risks of ISR and stent thrombosis—though reduced—persist in the setting of acute myocardial infarction (AMI). In this context, DCBs have emerged as a potential alternative strategy, particularly after restoration of coronary flow via balloon dilatation and thrombus aspiration, provided residual stenosis is minimal [51]. DCB therapy offers distinct advantages, including accelerated vascular healing and the preservation of native vasomotion, attributed to the absence of metallic struts and polymer-induced chronic inflammation [13] (Fig. 2, Ref. [37, 38]).

The Revascularization with Paclitaxel-Coated Balloon Angioplasty Versus Drug-Eluting Stenting in Acute Myocardial Infarction [REVELATION] trial (n = 120) studied the efficacy and safety of DCB vs. DES for ST-segment myocardial infarction (STEMI). The selected lesions were noncalcified and post-PTCA, with less than 50% stenosis before randomization to DCB vs DES. The results showed that DCB was non-inferior to DES after 9 months, when measured for fractional flow reserve (0.92 ± 0.05 in the DCB group and 0.91 ± 0.06 in the DES group; *p* = 0.27), and was safe and feasible for primary percutaneous intervention at 2-year follow-up [53, 54].

Gobić *et al*. [54] conducted a similar study, in which the safety and feasibility of DCB were assessed compared to DES in patients with STEMI. A total of 75 patients were enrolled in the study (37 in the DES group and 38 in the DCB group). After a 6-month follow-up, the study concluded that DCB is a safe strategy in patients with acute coronary syndromes as a primary intervention. There was no MACE in the DCB group vs. 5.4% of patients in the DES group (risk ratio = 5.13, 95% CI [0.25–103.42], *p* = 0.29).

A recent meta-analysis of randomized controlled trials (RCTs) evaluated the efficacy of DCB in patients with acute MI and found no significant difference in MACE rate (RR, 0.85; 95% CI 0.42 to 1.74; *p* = 0.66) and lower LLL (weighted mean difference (WMD), –0.29; 95% CI –0.46 to –0.12; *p* < 0.001) between DCB and stent groups, respectively [55].

These studies have shown that the DCB might be an effective strategy compared to stents, but careful attention is required in patient selection, adequate lesion preparation for optimal results, and to prevent bailout stent implantation [55].

In the context of NSTEMI, DCB usage was found to be non-inferior to bare metal stent (BMS) and DES based on the results of the PEPCAD-NTSEMI trial. The overall MACE rate was 6.7% compared to 14.2% (*p* = 0.11), and the TLF rate was 3.8% versus 6.6% (intention-to-treat, *p* = 0.53) in the DCB and DES/BMS groups, respectively. The data warrant further investigation and larger trials of DCB in this setting [56].

## 7. Diabetes Mellitus

Patients with diabetes mellitus (DM) are at heightened risk for cardiovascular events, with a significantly increased incidence of coronary revascularization. This subgroup is particularly prone to ISR and stent thrombosis, especially in small-caliber vessels, due to the presence of diffuse and complex lesions. The risk of restenosis and neointimal proliferation is inversely related to the diameter of the vessel. Furthermore, the inhomogeneous distribution of the drug associated with DES may promote local inflammation and platelet aggregation, contributing to ISR and stent thrombosis. In this context, DCBs represent a viable alternative therapeutic strategy [13].

A recent meta-analysis by Verdoia *et al*. [57] encompassing 10 studies with a total of 2026 patients evaluated the efficacy of DCB in patients with diabetes. At, a mean follow-up of 15 months, DCB use was associated with significantly lower rates of mortality [3.2% vs 4.9%; OR = 0.61 (0.38, 0.97), *p* = 0.04] and TLR [7.4% vs 10.9%; OR = 0.66 (0.44, 0.99), *p* = 0.05], along with a trend towards reduced MACE [13.6% vs 17.6%; OR = 0.79 (0.61, 1.04)] compared with DES [58]. Similar findings were reinforced by a meta-analysis by Li *et al*. [58], which compared the short-term outcomes of DCB with those of DES in small vessels and found that DCB is superior to DES in terms of efficacy and safety [59]

Several prospective studies by Pan *et al*. [59], Benjamin *et al*. [60], and the EAST BOURNE registry [61] evaluated the safety and feasibility of DCB usage in diabetic events. They found higher TLR and TLF rates, similar to higher MACE rates observed in non-diabetics (Table 5, Ref. [59, 60, 61]).

**Table 5.** **Summary of trials and studies on DCB use in diabetes mellitus**.

Study/Trial name	Number of patients (n)	Study design	Treatment arm	Clinical follow-up	Conclusion
Pan *et al*. 2021 [59]	2306	Prospective observational; 2021	Non-DM (578) vs DM (578)	TLR 1.90% (non-DM) vs. 4.15% (DM); OR, 2.233	Higher TLF, TLR, and similar MACE in the diabetes group
Benjamin *et al*. 2021 [60]	1198	Prospective observational	Non-DM (768) vs DM (430)	TLF 1.4% (non-DM) vs 3.9% (DM) @1 year	Higher TLF and similar MACE in the diabetes group
EAST BOURNE. 2023 [61]	2083	Prospective subgroup analysis	Non-DM (1219) vs DM (864)	TLR 4.3% (non-DM) vs 6.5% (DM) MACE 8.1% (non-DM) vs 11% (DM)	Higher TLR and MACE observed in diabetics vs. non-diabetics

DM, diabetes mellitus.

Based on the results, DCB use in patients with diabetes mellitus needs further validation with large-scale trials.

## 8. Chronic Total Occlusion

The application of DCBs in chronic total occlusion (CTO) lesions offers several advantages, including the preservation of vasomotion, promotion of positive arterial remodeling, prevention of undersized stent placement and associated complications, and avoidance of full metal jacket deployment. However, the existing literature on DCB utilization in CTO interventions remains limited. A recent meta-analysis by Natarajan *et al*. [62] encompassing 10 studies (5 comparative and 5 single-arm) with a total of 1695 patients, demonstrated no significant differences in major MACE, TLR, and target vessel revascularization (TVR) rates among DCB, DES, and hybrid strategies in both *de novo* and in-stent CTO cohorts. Despite the paucity of robust evidence, the use of DCB, either as a standalone therapy or in combination with DES, appears to be a safe and viable alternative in CTO percutaneous PCI, thereby warranting further large-scale randomized controlled trials. Nevertheless, the precise role of DCB in cases involving subintimal entry remains undefined and necessitates additional investigation.

## 9. Future Directions

Beyond their well-established role in treating ISR, DCBs have shown promise as an alternative to DES across a range of coronary lesions in various challenging clinical and technical scenarios, as discussed above. At this juncture, there is a compelling need for large-scale, randomized studies to define further the role of DCBs in both specific patient subsets and the broader management of CAD, including acute coronary syndromes—whether as a complement to or a substitute for DES. While paclitaxel-coated balloons currently represent the cornerstone of DCB therapy, ongoing innovation is focused on next-generation agents and advanced delivery platforms that may enhance safety, drug transfer efficiency, and long-term outcomes. Challenges related to sirolimus’s relatively low lipophilicity—which limits vascular wall penetration and drug retention—remain an area of active investigation, prompting the development and evaluation of alternative delivery technologies [38].

The novel sirolimus-eluting balloon (Selution; Cordis) has emerged as a promising alternative for both *de novo* coronary disease and ISR. Recently presented results from the SELUTION DeNOVO randomized trial demonstrated the non-inferiority of DCB with provisional stenting compared with DES, with a 1-year primary endpoint of target vessel failure (5.3% vs. 4.4%; *p* = 0.02) [63, 64]. Current ongoing randomized trials, including TRANSFORM II, are aimed at looking into sirolimus-coated balloon versus drug-eluting stent in *de novo* coronary lesions [38].

## 10. Conclusion

**Fig. 3.**
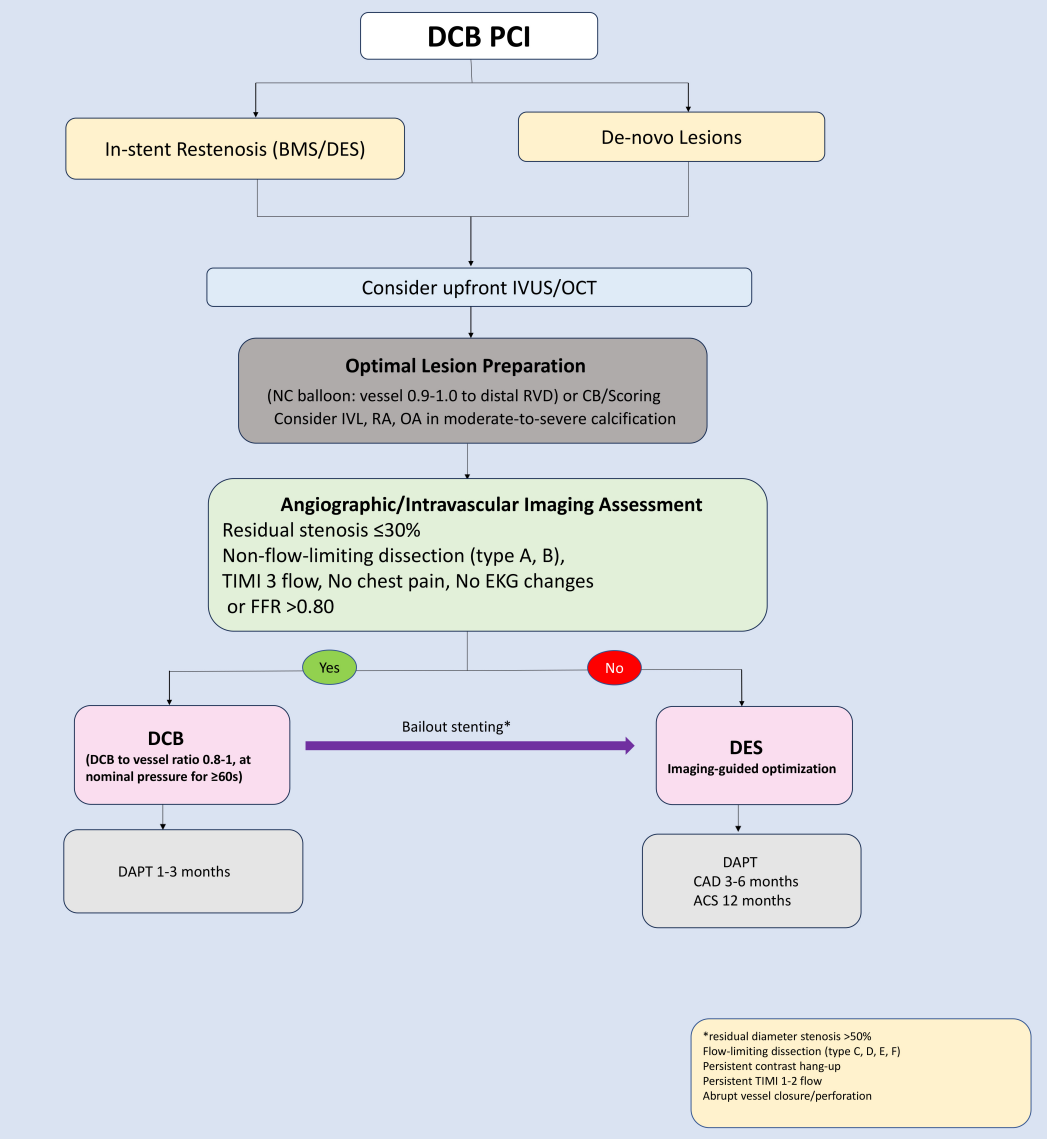
**Expert opinion–based practical algorithm for DCB-only PCI [37, 38]**. RVD, reference vessel diameter; CB, cutting balloon; FFR, fractional flow reserve; DAPT, dual antiplatelet therapy; SAPT, single antiplatelet therapy.

DCBs are rapidly emerging as an effective and safe therapeutic strategy for patients with coronary artery disease. Optimal lesion preparation remains the cornerstone of successful DCB application (as shown in Fig. 3, Ref. [37, 38]). Currently, DCBs are FDA-approved for ISR in the USA, with use in *de novo* lesions remaining off-label. While paclitaxel-coated balloons have shown a signal for increased mortality in endovascular peripheral interventions [65], this has not been observed in coronary applications, as confirmed in multiple randomized trials, including BASKET-SMALL 2 [28] and DEBUT [52], which demonstrated no increased mortality in coronary settings. The 3-year outcomes of the REC-CAGEFREE I trial provided valuable insights, and the ongoing follow-up, continuing up to ten years, will further strengthen the data [22]. The positive results from the large-scale sirolimus-coated SELUTION DeNovo trial [63, 64] represent a pivotal turning point that may reinvigorate the DCB field. Future trials are expected to address the knowledge gaps, refine the pharmacological and technical aspects of DCB technology, and expand its application beyond ISR toward a broader stentless interventional paradigm in coronary revascularization.

## Abbreviations

DCB, drug-coated balloon; DES, drug-eluting stent; MACE, major adverse cardiac event; STEMI, ST-segment elevation myocardial infarction; TLR, target lesion revascularization; TLF, target lesion failure; CTO, chronic total occlusion; SVD, small-vessel disease; POBA, plain old balloon angioplasty; PCB, paclitaxel-coated balloon; LLL, late luminal loss; TVR, target vessel revascularization; IVUS, intravascular ultrasound; OCT, optical coherence tomography; RVD, reference vessel diameter; CB, cutting balloon; FFR, fractional flow reserve; DAPT, dual antiplatelet therapy; SAPT, single antiplatelet therapy; BMS, bare-meta stent; WMD, weighted mean difference.
